# Supplementary figures and images for: Prospects and limitations of expansion microscopy in chromatin ultrastructure determination
Source: Chromosome Res. 2020 Sep 17;28(3):355–68. doi: 10.1007/s10577-020-09637-y (PMC7691311; doi:10.1007/s10577-020-09637-y)

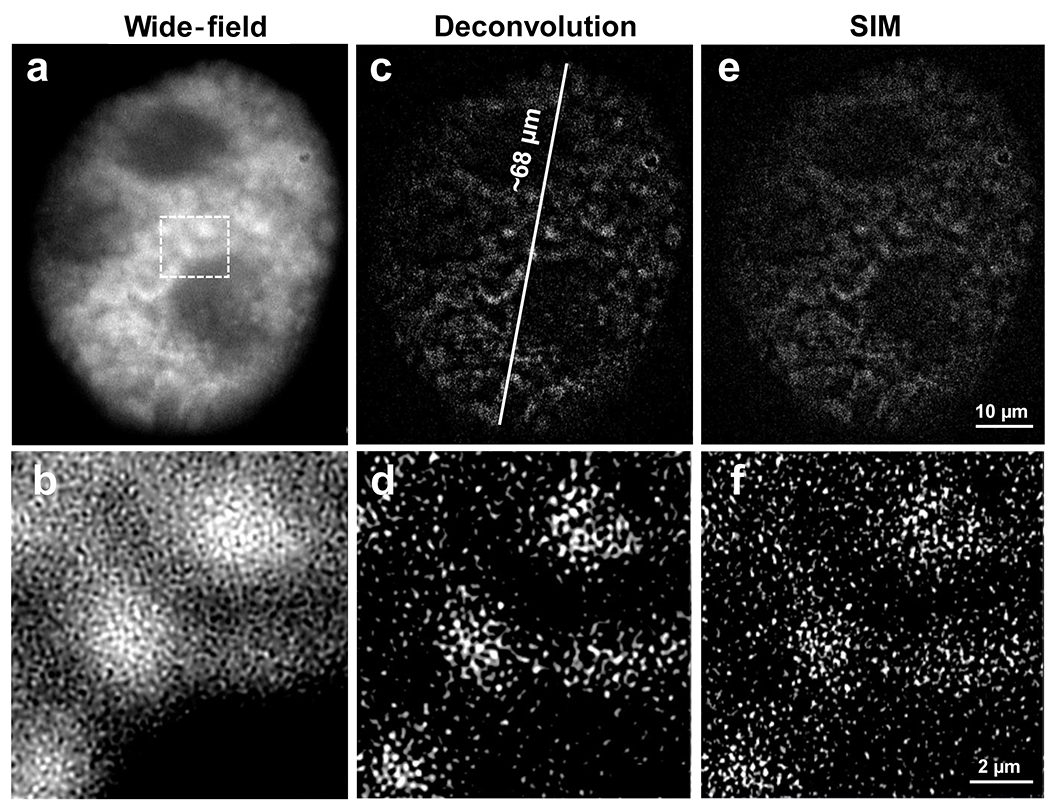

Supplement: Supplementary file 2 — The chromatin ultrastructure is impaired by heat denaturation applying ExM protocol variant 2A (see Table 1) to achieve complete expansion, as especially demonstrated by SIM of the magnified selected region (dashed rectangle). The expanded nucleus was imaged by WF (a, b), deconvolution (c, d) and SIM (e, f). Global chromatin was stained by DAPI. Global chromatin was stained by DAPI. (PNG 731 kb) [file 10577_2020_9637_Fig7_ESM.png]

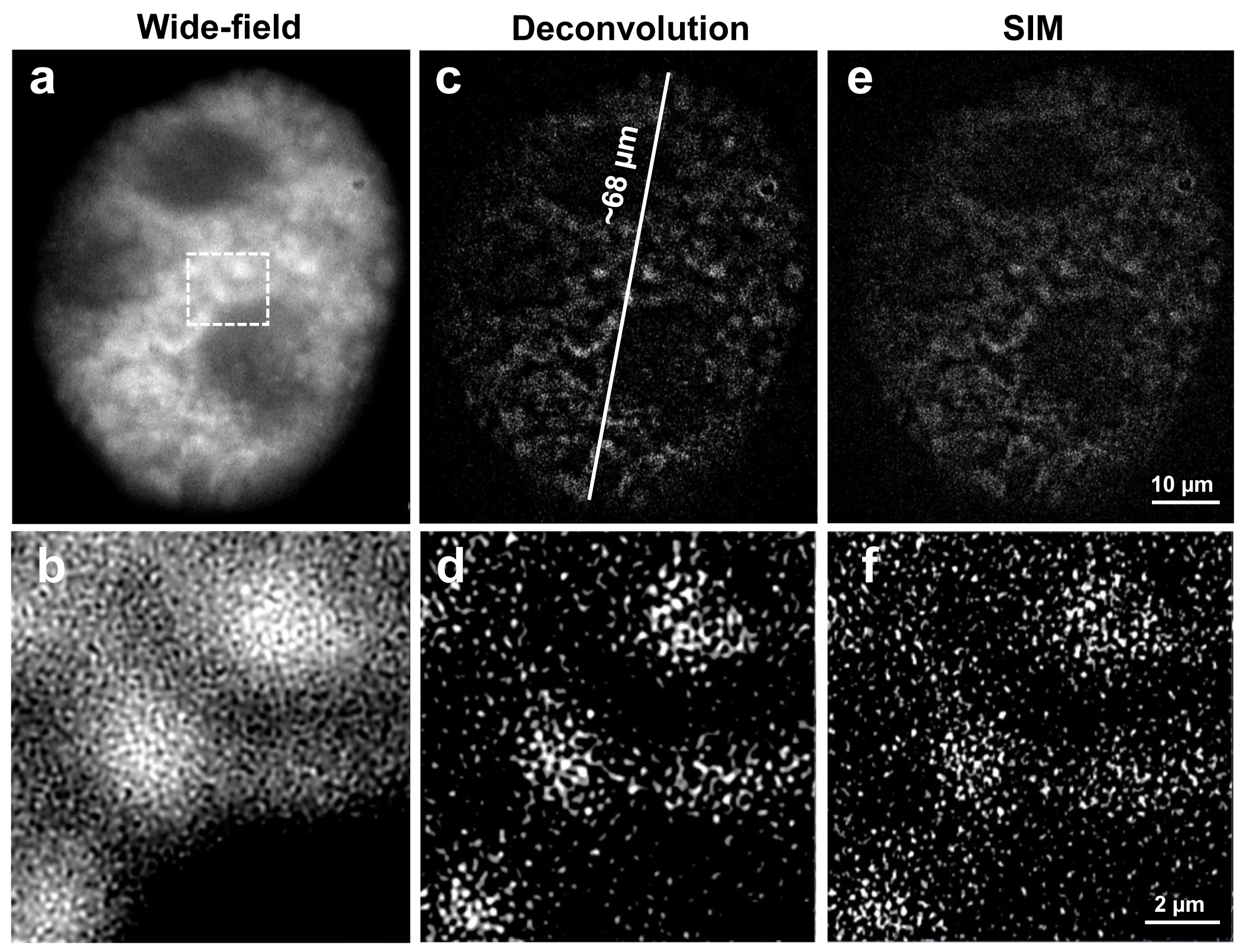

Supplement: Supplementary file 3 — High Resolution Image (TIF 2.69 mb) [file 10577_2020_9637_MOESM2_ESM.tif]

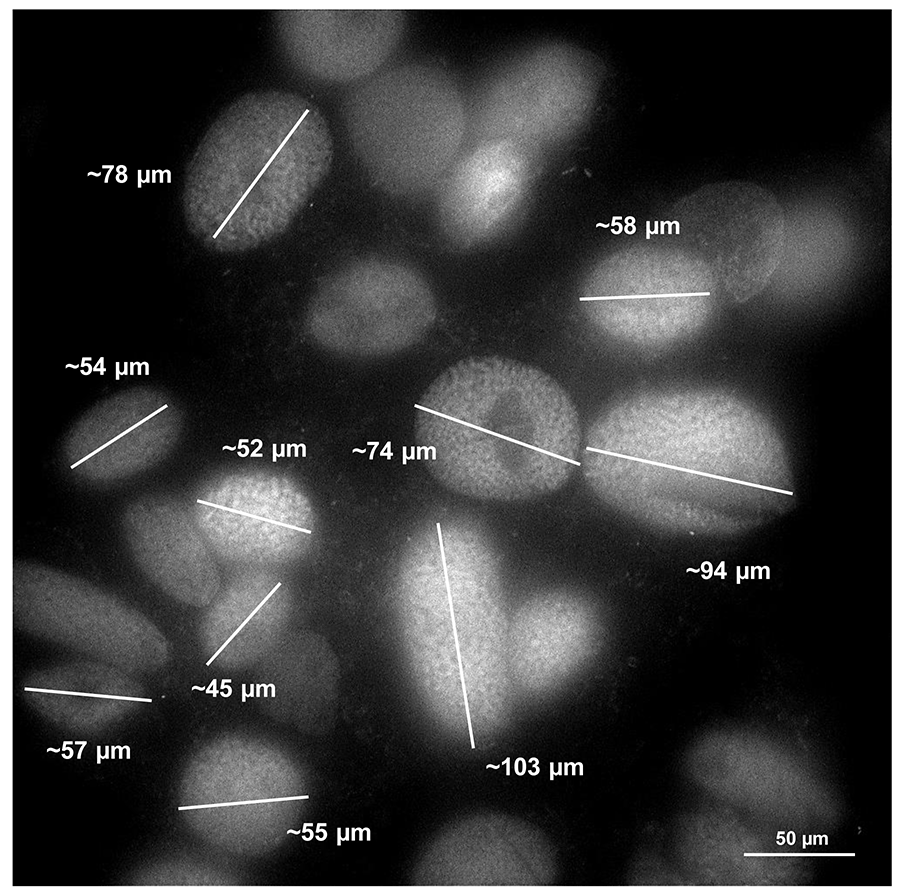

Supplement: Supplementary file 4 — Mixture of different nuclei from root tips after expansion acquired by a 20×/0.8 objective. The nucleus population originates from different cell cycle stages (G1, S, G2) and tissue layers of the root tip. The differences in DNA content influence the size of the nuclei and thus a high size variability is also present after expansion. (PNG 2.30 mb) [file 10577_2020_9637_Fig8_ESM.png]

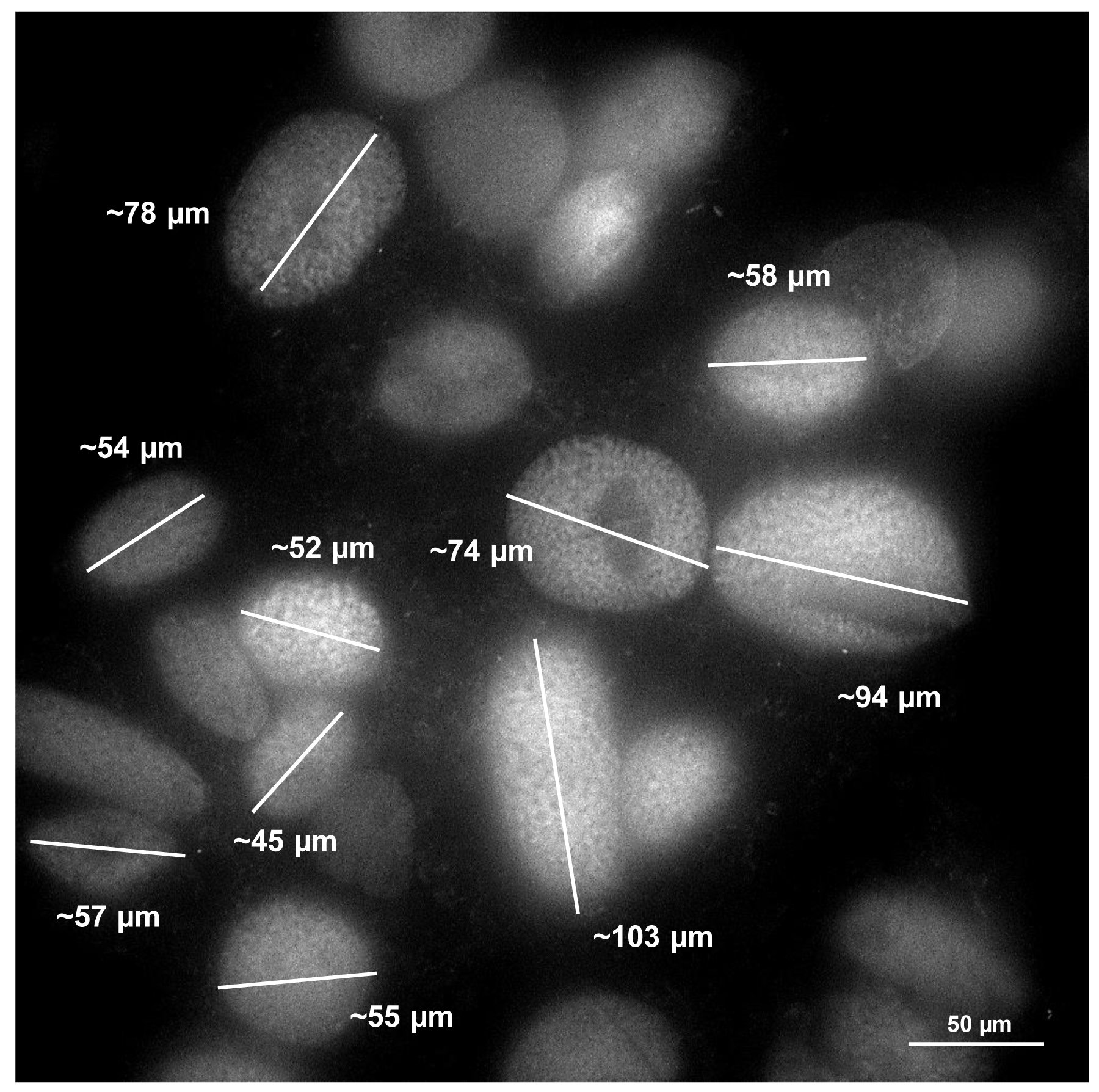

Supplement: Supplementary file 5 — High Resolution Image (TIF 2.51 mb) [file 10577_2020_9637_MOESM3_ESM.tif]

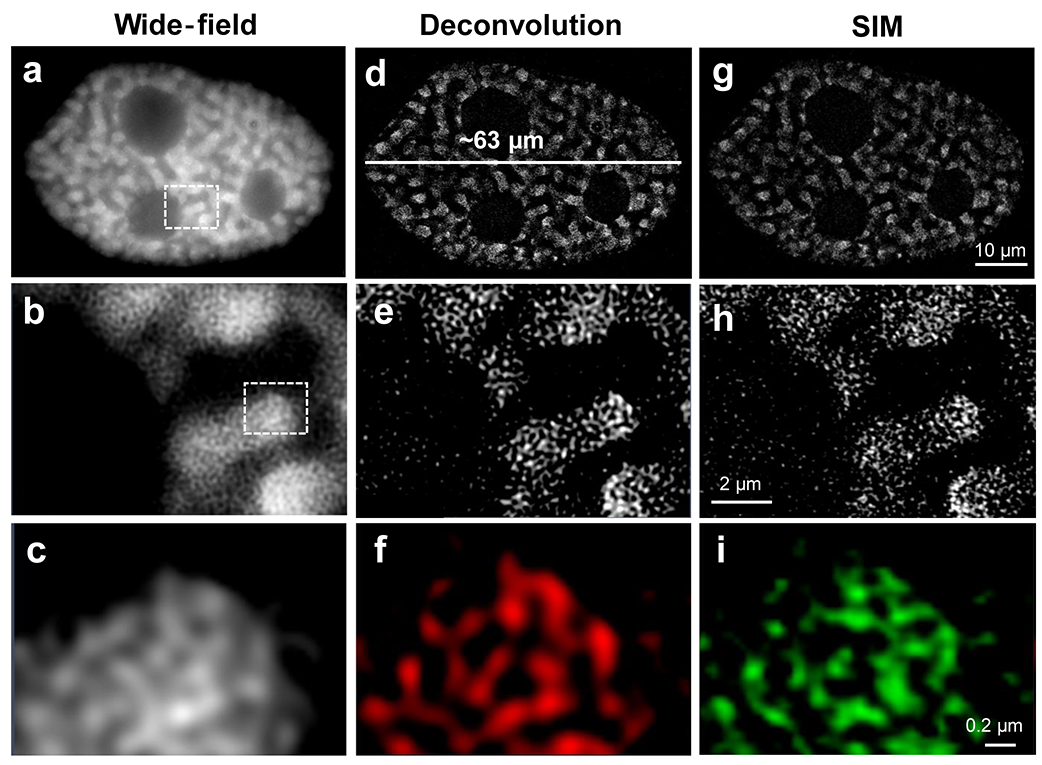

Supplement: Supplementary file 6 — The chromatin ultrastructure is mainly preserved after denaturation with NaOH applying ExM protocol variant 3A (see Table 1) to achieve complete expansion. By WF (a, b, c), deconvolution (d, e, f) and SIM (g, h, i) identical chromatin ultrastructures labelled by DAPI become visible as especially demonstrated at the highest magnification (bottom panel) of the magnified selected regions (dashed rectangles). (PNG 514 kb) [file 10577_2020_9637_Fig9_ESM.png]

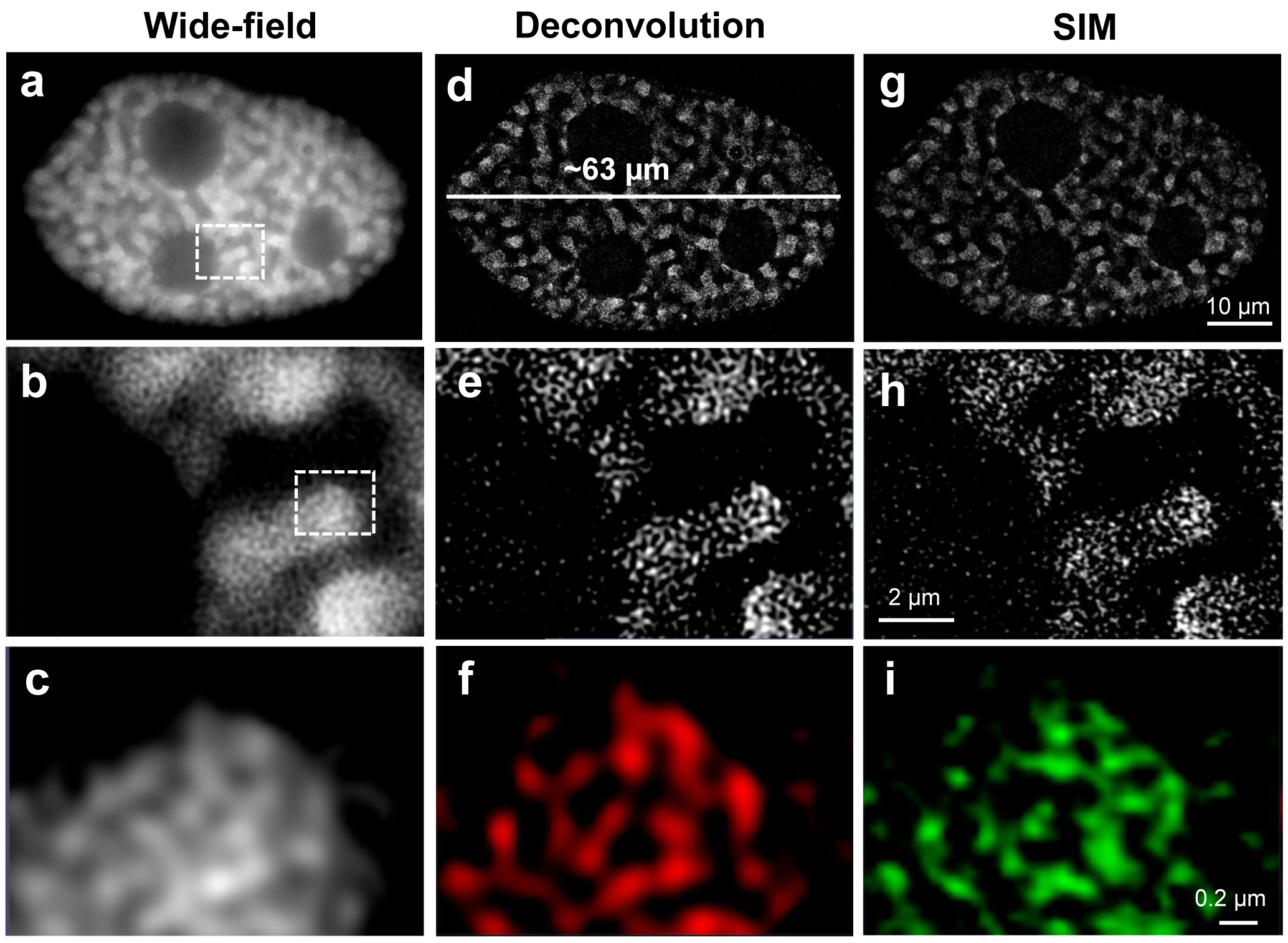

Supplement: Supplementary file 7 — High Resolution Image (TIF 1.81 mb) [file 10577_2020_9637_MOESM4_ESM.tif]

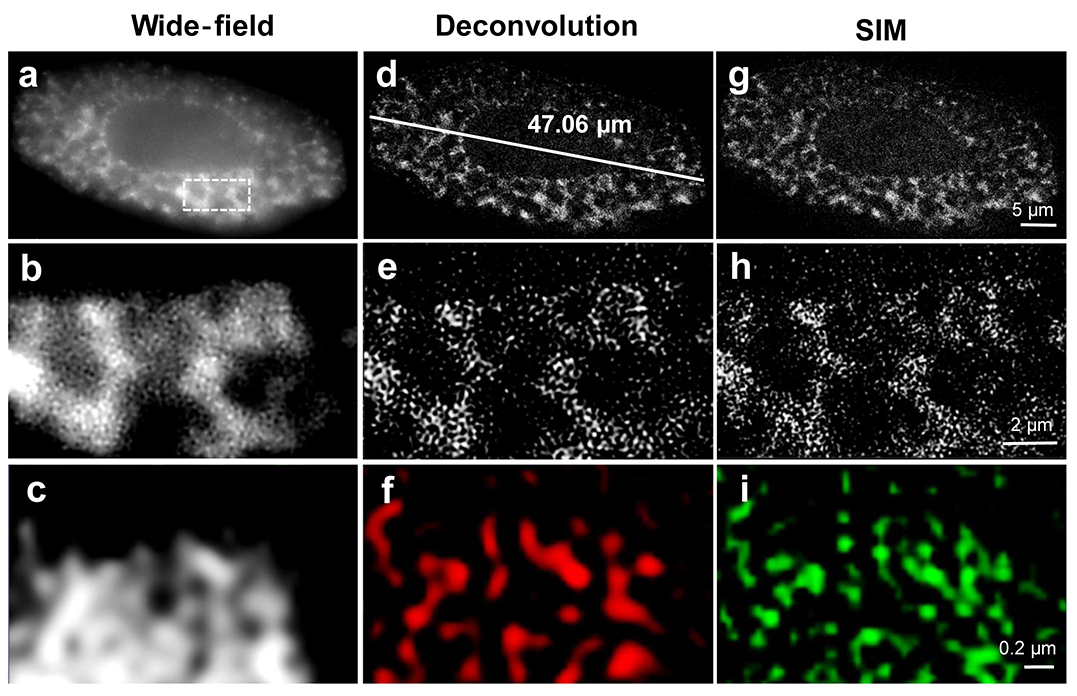

Supplement: Supplementary file 8 — The fixation with FA/GA and post-fixation with GA (ExM protocol variant 12A, see Table 1) delivered less expanded nuclei than the FA/AA fixation. The chromatin domains visible in WF (a-c). deconvolution (d-f ) and SIM (g-i) showed that the native network-like chromatin structure was impaired. (PNG 565 kb) [file 10577_2020_9637_Fig10_ESM.png]

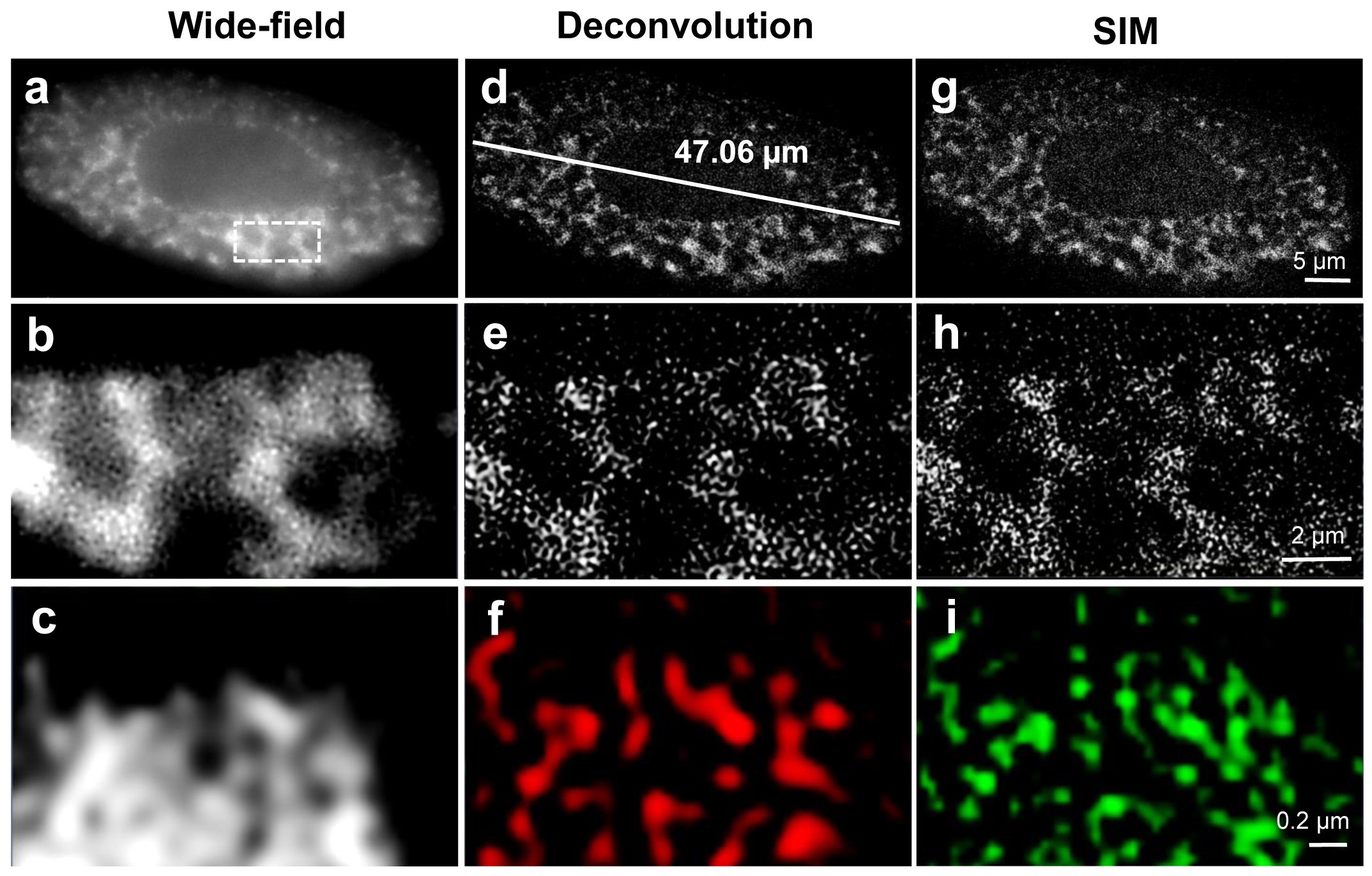

Supplement: Supplementary file 9 — High Resolution Image (TIF 2.00 mb) [file 10577_2020_9637_MOESM5_ESM.tif]

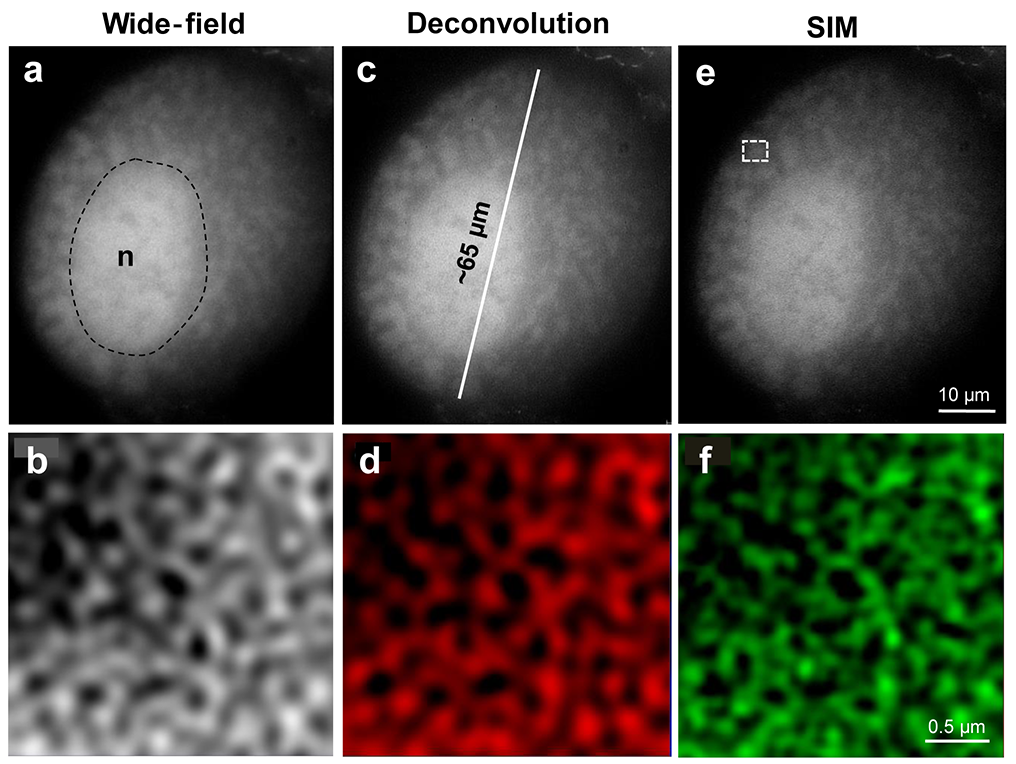

Supplement: Supplementary file 10 — Although proteinase K treatment (protocol variant 2B in Table 1) does not impair the chromatin ultrastructure the digestion induces unspecific nucleolus (n) labelling. By WF (a, b), deconvolution (c, d) and SIM (e, f) imaging, especially at high magnification (bottom panel) of the selected region (dashed rectangle) similar chromatin structures labelled by DAPI become visible. (PNG 523 kb) [file 10577_2020_9637_Fig11_ESM.png]

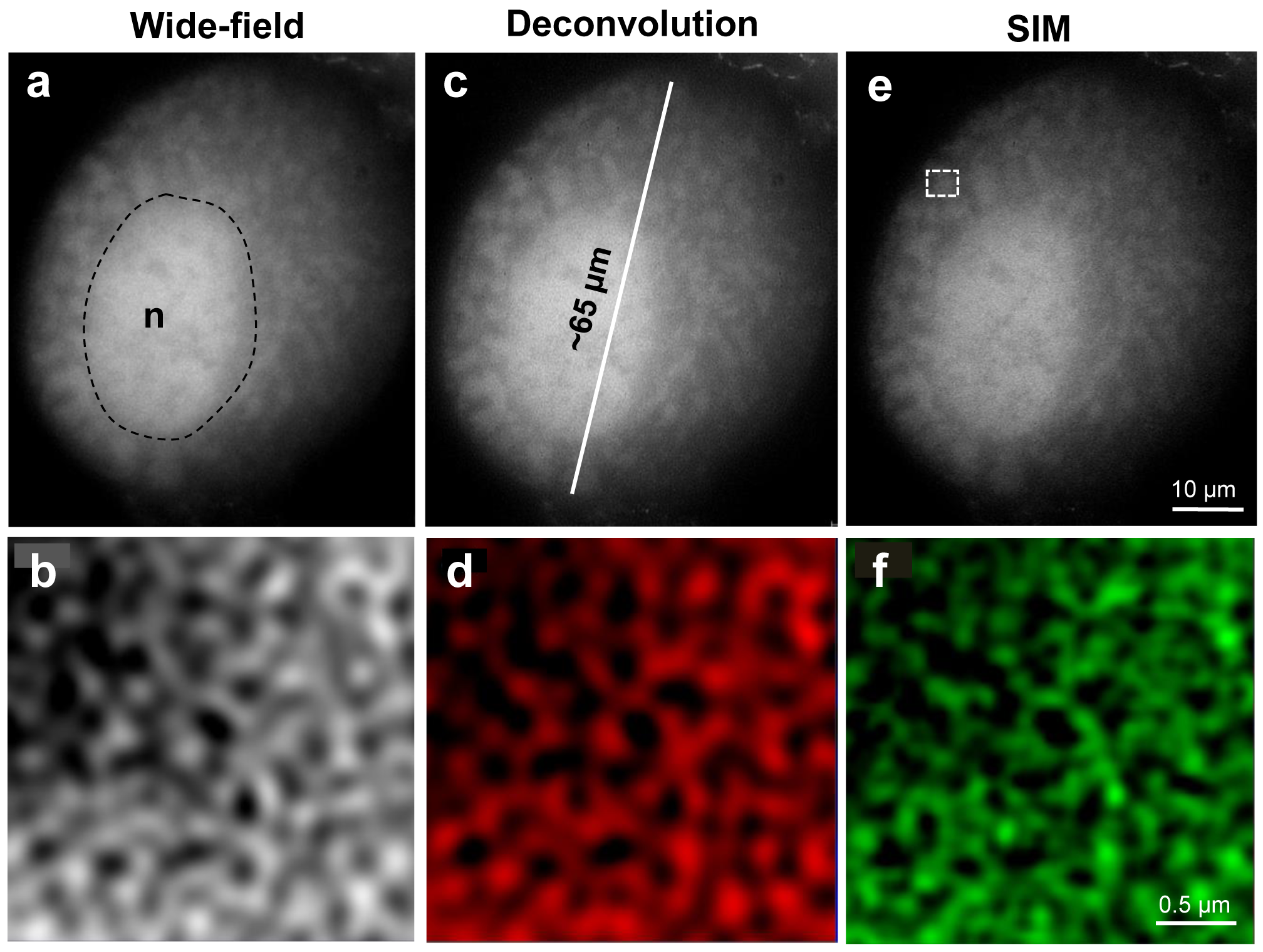

Supplement: Supplementary file 11 — High Resolution Image (TIF 1.96 mb) [file 10577_2020_9637_MOESM6_ESM.tif]
